# Building Capacity and Training for Digital Health: Challenges and Opportunities in Latin America

**DOI:** 10.2196/16513

**Published:** 2019-12-18

**Authors:** Walter H Curioso

**Affiliations:** 1 Department of Biomedical Informatics and Medical Education University of Washington Seattle, WA United States

**Keywords:** digital health, capacity building, training program, education, public health, telehealth, Peru, Latin America

## Abstract

Tackling global health challenges demands the appropriate use of available technologies. Although digital health could significantly improve health care access, use, quality, and outcomes, realizing this possibility requires personnel trained in digital health. There is growing evidence of the benefits of digital health for improving the performance of health systems and outcomes in developed countries. However, significant gaps remain in resource-constrained settings. Technological and socio-cultural disparities between different regions or between provinces within the same country are prevalent. Rural areas, where the promise and need are highest, are particularly deprived. In Latin America, there is an unmet need for training and building the capacity of professionals in digital health. This viewpoint paper aims to present a selection of experiences in building digital health capacity in Latin America to illustrate a series of challenges and opportunities for strengthening digital health training programs in resource-constrained environments. These describe how a successful digital health ecosystem for Latin America requires culturally relevant and collaborative research and training programs in digital health. These programs should be responsive to the needs of all relevant regional stakeholders, including government agencies, non–governmental organizations, industry, academic or research entities, professional societies, and communities. This paper highlights the role that collaborative partnerships can play in sharing resources, experiences, and lessons learned between countries to optimize training and research opportunities in Latin America.

## Introduction

Digital health has made a wide range of technologies (eg, the use of computers, tablets, and mobile phones) readily available around the globe, and could contribute significantly to both improving the quality of health care for populations and enabling better health outcomes [1]. In this viewpoint, the term digital health could be used synonymously with electronic health (eHealth) and mobile health (mHealth).

Although there is growing evidence of the benefits of digital health for strengthening health systems and improving health outcomes in developed countries, significant gaps remain in resource-constrained settings. Technological and socio-cultural disparities between different regions or provinces within the same country are prevalent. Rural areas, where the promise and need are highest, are particularly deprived.

Latin America is still recognized as the most unequal region in the world [2]. According to the 2019 Global Multidimensional Poverty Index report by the United Nations Development Programme and the Oxford Poverty and Human Development Initiative, 39 million people in Latin America experience multidimensional poverty [3]. Latin America is a region with particular local characteristics related to economy, employment, housing, education, cultural context, and health care [4].

Information and communication technologies can help reduce those inequalities in Latin America, being essential tools to promote innovation and knowledge generation in societies, especially to disadvantaged groups and rural areas. Latin American countries must properly incorporate information and communication technologies to address the health needs of their population. In October 2019, the 57th Directing Council of the Pan American Health Organization (PAHO), through Resolution CD57/9, adopted the Plan of Action for Strengthening Information Systems for Health 2019-2023 [5] to ensure better collection and management of data. This was done to inform decision-making, policy development, monitoring, and evaluation. This highlights the need for strengthening resource capacity for the proper implementation of health information systems, considering the context, needs, vulnerabilities, and priorities of countries [5]. Objective 42.2 is related to improving human resource training in all aspects of health information systems, and is the [5]:

Number of countries and territories with ongoing professional training strategies or digital literacy programs for the use of new technologies.

Excepting the countries of the Americas that have made substantial progress in improving health information systems [5], there are still many technological, organizational, and socio-cultural challenges in Latin America. These challenges include poor infrastructure, low Internet connectivity, inequalities in access to electricity, and adult illiteracy [6]. This lack of efficient health innovation projects and programs has been recognized as one of the main challenges of the health sector in the region [7]. On top of that, the World Health Organization (WHO) and the International Telecommunication Union identified the lack of qualified and experienced professionals in digital health as one of the main barriers for digital health application at the global level [8]. In general, doctors are unevenly distributed across Latin American countries, and most health care professionals are concentrated in urban areas [7].

Therefore, we need a skilled workforce not only to understand health care and information and communication technologies, but also to implement effective digital health systems that can achieve national, regional, and local goals. This is especially important when considering the socio-economic, socio-cultural, and organizational challenges involved. This viewpoint paper aims to present a selection of experiences in building resource capacity for digital health in Latin America to illustrate a series of challenges and opportunities for strengthening digital health training programs in resource-constrained environments.

## The Need for Strengthening the Digital Health Workforce in Latin America

The need for training and building the capacity of health care professionals in digital health remains one of the most significant public health challenges in Latin America [9]. Digital health training and research programs in this region are scarce [10] and poorly documented. However, some remarkable initiatives and programs have been implemented in Latin America. For example, the PAHO has carried out essential actions regarding digital health in the region, which included not only capacity-building activities but also research. These activities included regional workshops on digital health, the development of knowledge networks and technical guidelines, the promotion of information sources on the internet, the establishment of digital health sustainability models, the support for the development of electronic health records, the promotion of standards on health data and related technologies that ensure exchange of information, the promotion of mobile devices to improve health, and the improvement in quality of care through telemedicine [11].

Moreover, the PAHO has developed a series of virtual courses such as: “eHealth for Managers and Decision Makers,” the “Virtual course on properly completing death certificates,” and “Access and Use of Scientific Information on Health.” All these courses are available through the Virtual Campus of Public Health. With those initiatives, thousands of health workers have already been trained throughout the region [12].

## Experiences for Building Capacity in Digital Health in Latin America

A proper health workforce trained in digital health will strengthen health systems and ensure adequate service delivery. There is growing evidence around the world of the value that well-trained personnel in digital health can offer to tackle global health challenges [13], including evidence from Latin America [14,15]. Latin American countries have carried out a wide range of digital health initiatives, including educational initiatives and training programs for digital health resource capacity building [5,10,16-19]. Despite the discontinuity of several projects and initiatives, many collaborative courses and research projects related to digital health have been developed between international institutions promoting North-to-South, South-to-North, and South-to-South collaborations [5,10,20-24].

The Informatics Training for Global Health (ITGH) program from the Fogarty International Center has played a significant role in expanding the informatics workforce based on the health and informatics needs of countries. Over the years, the ITGH has supported informatics research training in low- and middle-income country institutions in partnership with US institutions and investigators and has funded several collaborative research and training programs in Latin America [10,13,15]. 

The American Medical Informatics Association (AMIA)’s Global Partnership Program (GPP), funded by the Bill and Melinda Gates Foundation, aims to develop project-centric approaches to training in developing countries to expand local resource capacities and to promote collaboration [25]. As part of the meetings conducted with AMIA GPP committee members, several Latin American experts on digital health actively participated and contributed to the discussions and activities [26].

There is also “Regional Protocols for the Formulation of Public Telehealth Policies for Latin America,” funded by the Inter-American Development Bank, which involved 16 Latin American countries and identified that the training of professionals in telehealth was a priority for its development in the region [16]. To contribute to the development of telehealth in the region an international telehealth training course (via long-distance education) was conducted with the participation of Latin American universities, the PAHO, and the United Nations Economic Commission for Latin America and the Caribbean, [16].

It is essential to highlight that Brazil once hosted the oldest resident informaticist program in Latin America, at the Hospital das Clínicas da Faculdade de Medicina da Universidade de São Paulo, from 1988-1997 [27]. The clinical pathology service had a 2-year clinical informatics residency mainly focused on hospital software project management, the design of electronic health records, and epidemiological information technologies [27]. Brazil also launched electronic learning programs for the National Telehealth Program and in partnership with the Open University of the Unified Health System [10], and they also promoted the development of nursing education programs with nursing informatics topics [10].

It is also important to point out that the Department of Health Informatics at Hospital Italiano de Buenos Aires, in Argentina, created in 2001, developed a 4-year residency program that has been accredited by national authorities as a “bridge” between health care and information technology [10]. The curriculum includes two years in internal medicine, computer science, health care information systems, electronic health records management, epidemiology, knowledge-based databases for clinical terminology and standards, biostatistics, and decision-making theory [10]. Likewise, the Hospital Italiano de Buenos Aires developed courses for digital literacy oriented towards information retrieval and other similar disciplines. A Spanish version of the AMIA’s 10×10 initiative was developed in collaboration with Oregon Health and Science University’s Department of Medical Informatics and Clinical Epidemiology [10].

Training programs on digital health in Latin America should consider the results from a survey conducted in 2011 by Blas et al from the Andean Global Health Informatics Research and Training Center [21], which surveyed 142 experts from 11 countries regarding the need for health informatics training. The top-ranked courses were: “Introduction to Biomedical Informatics,” “Data Representation and Databases,” “Mobile Health,” and “Security, Confidentiality, and Privacy.” The research topics reported as a priority were: evaluation of health information systems, policy in health informatics, interoperability and standards, evidence-based decision making in informatics, rural telemedicine, mobile health, electronic health records, sequence analysis and gene finding, tele-education, and the analysis of cost-effectiveness in biomedical informatics [21].

## Challenges and Opportunities for Strengthening Training Programs and Initiatives in Latin America

### Overview

A remarkable 2003 review paper by Rodrigues, published at the Journal of Medical Internet Research [28], and entitled “eHealth in Latin America and the Caribbean: Development and Policy Issues,” described trends and issues regarding the deployment of electronic health solutions in Latin America and the Caribbean. Since people are central to the value-added creation of digital health services, skilled and committed human resources were identified as an essential component for the deployment of the digital health ecosystem in Latin America [28]. Therefore, Latin American countries should invest in digital health training programs to overcome main health care challenges. Rodrigues proposed that systems professionals, technology products, services providers, and project teams must have superior skill levels and experience. He also proposed that efficient and robust government and academic institutions should be committed to investing in education, scientific and technological development, and public services [28]. Some of the critical things that Latin American governments should focus on developing and implementing include developing a national vision, mission, and plan of action for the public and private sectors, and the promotion of education, training, and national planning capacity in information systems and technology [28].

Latin America faces several challenges regarding digital health. For example, telehealth has not yet been scaled up broadly at the national and regional level due to organizational, physical, and technological challenges in Latin America [29]. Moreover, the cultural context and core cultural values in the health care process play a significant role and could have important implications for digital health adoption [29]. Data sharing among health professionals also remains a limitation due to the restricted use of technology for communication, training, and decision-making [30].

Some risks for training programs on digital health might include lack of clarity about the scope of the training program and the responsibilities, insufficient capacity to carry out the program and develop an appropriate curriculum, inability to retain skilled staff, participation of experts with very little or no knowledge of the local needs and socio-cultural issues, dependency on external support, and insufficient planning for sustainability [31]. One of the key points to address those risks effectively is to establish cooperative partnerships that need to be addressed and planned [32,33].

In 2008, the Rockefeller Foundation launched a month-long conference series called “Making the eHealth Connection: Global Partnerships, Local Solutions,” held at the Rockefeller Foundation Bellagio Center in Italy. It hosted 200 global experts on digital health from 34 countries, including Latin American researchers and experts [34]. The report from the conference series entitled “From Silos to Systems” [35] presented a chapter on “eHealth Capacity Building,” which defined a vision with three objectives: (1) create an international network of eHealth informatics practice, education, training, policy, and research; (2) educate government leaders about the importance of eHealth capacity and informatics to help develop national health goals, economic goals, and to cultivate and sustain support for eHealth resource capacity growth and informatics activities; and (3) develop a blueprint for initiating and executing activities in resource-poor countries to rapidly create eHealth initiatives.

The mHealth Alliance was seeded at the conference and has promoted summits, technical working groups, and other informal networking activities to strengthen links among Latin American researchers and global digital health stakeholders [36]. In this sense, there are tremendous opportunities to boost the promotion of research and training through networking between countries in Latin America and beyond.  Some experiences of collaborative initiatives in Latin America that could be fostered and even applied to other countries are described below.

### The AMAUTA Program and the QUIPU Network

In 1999, the AMAUTA Global Training in Health Informatics program was developed to train Peruvian health care professionals in the application of informatics to health [37]. This collaborative program was an institutional partnership that involved Universidad Peruana Cayetano Heredia, Universidad Nacional Mayor de San Marcos, and the University of Washington, Seattle, Washington, with the support of the Fogarty International Center and the National Institutes of Health. The AMAUTA program achieved considerable success in the development and institution of informatics research and training programs in Peru [37]. Courses and projects supported by the AMAUTA program led to the development of sustainable training opportunities for biomedical informatics in Peru [38] and contributed to their development in other low- and middle-income countries.

This program later expanded to other Latin American countries through the QUIPU Center, which worked to promote South-to-South collaborations [37]. As part of The Andean Global Health Informatics Research and Training Center, the QUIPU network was created in 2010. The objectives of QUIPU were to: (1) develop and implement short- and long-term training opportunities in biomedical informatics for global health in the Andean region; (2) engage new researchers in the Andean region to research health informatics and bioinformatics; and (3) expand and consolidate a research network in the Andean region, promoting South-to-South collaboration and collaborative initiatives with United States–based universities and institutions [39]. This Center has developed several face-to-face and virtual courses [39] and has supported the establishment of a diploma and a Master’s program on Biomedical Informatics [40], the first of such training programs in Peru.

### Federation of Health Informatics for Latin America and the Caribbean

The International Medical Informatics Association (IMIA) has a Federation of Health Informatics for Latin America and the Caribbean called IMIA-LAC. As of January 2018, 12 medical informatics societies were members of the Federation [41]. The regional activities of IMIA-LAC have been scarce, but it is desirable to strengthen regional ties with close links to universities and societies at the international level [42].

### The Central American Health Informatics Network

The Central American Health Informatics Network (RECAINSA), established in 2013, is a network of volunteers working in technology-related public and private health services [43]. One of its objectives is to support national digital health strategies, including generating networking spaces for the exchange of experiences and best practices. Another objective of RECAINSA is to strengthen digital health governance through legal and strategic frameworks through the participation of key stakeholders within the health sector [43]. RECAINSA also promotes the strengthening of human resources in universities, certified training centers, and other specialized organizations [43].

### The Latin American and Caribbean Network for Strengthening Health Information Systems

The Latin American and Caribbean Network for Strengthening Health Information Systems (RELACSIS), established in 2010 [44], is a community of best practices to improve data quality. It is made up of members from over 25 countries, comprising both health information users and managers [11]. RELACSIS promotes the strengthening of information systems for health through face-to-face and virtual courses (eg, ICD coding, complete death certificates), discussion forums, working groups, webinars, software solutions, and other low-cost tools. In this context, PAHO has organized several virtual seminars with experts to learn about the current status of electronic health records in the region [11].

### Ibero-American Network of Mobile Technology and Health

The Ibero-American Network of Mobile Technology and Health (CYTED-RITMOS) is an international network led by the Open University of Catalonia and composed of 17 research groups from six countries (Argentina, Bolivia, Chile, Colombia, Ecuador, and Spain), the PAHO/WHO, Doctors Without Borders, Telefónica, the Mobile World Capital Barcelona Foundation, and the University of Michigan. This network aims to promote research and development of mHealth in Latin America [45]. In October 2015, the International RITMOS Workshop took place in Barcelona, intending to determine the priority areas of Latin America in which research, development, and innovation projects in mHealth could be developed [45].

### Others

Several other international organizations are involved with a vast number of professionals who work on digital health projects and services in Latin America. Health Level Seven Latin America (HL7 LATAM) is a regional coordinating body that promotes the use of interoperability standards in Latin America to improve health care institutions in the region [46]. The current activities of HL7 LATAM are available through their website and on their social networks (HL7 LATAM). Healthcare Information and Management Systems Society (HIMSS) Latin America is an international organization focused on promoting initiatives and events on leadership, community building, and professional development [47]. Their main initiatives are holding conferences in Costa Rica, Brazil, and Chile, and supporting Latin American countries through the digital transformation of their health services [47].

There are also collaborative partnerships, such as the e-Government Cooperation Center (eGCC) (launched as a cooperative project between Korea and partner countries, such as Peru), which has supported digital health resource capacity building, the exchange of information technologies, and joint research projects. The Ministry of Interior and Safety and The National Information Society Agency of the Republic of Korea have worked together to provide support to international countries in e-Government related areas, such as smart cities and telemedicine [48].

Finally, the World Health Organization has established a new roster of experts in various areas related to digital health, which shows promise for boosting collaboration between experts from Latin America and abroad. Its goal is to share expertise and lessons learned regarding the development, implementation, and evaluation of capacity-building programs for digital health [49].

## Final Remarks

Training programs on digital health in Latin America should incorporate the local context, consider the local needs, and be sensitive to the local economic, social, cultural, and organizational factors. There is a need to develop innovative intercultural and multilingual educational tools and solutions according to the health needs of populations, especially in underserved areas.

Given the limited resources in the Latin American region, the creation and strengthening of the existing networks of public and private institutions with academic centers who have experience in digital health training and research should be promoted. They should also seek to work in close collaboration with local, regional, and national governments. Partnerships with developed institutions as part of a well-planned strategy have shown encouraging results. It is essential to point out that each partnership is unique, and each program has to ensure that the curriculum is relevant to address local needs and country priorities.

Training programs should promote and encourage international exposure and internships or clerkships in excellent digital health centers around the world. Potential outcomes of those experiences could be research projects conducted in a wide variety of topics, such as electronic medical records, telemedicine, mobile health, artificial intelligence, the internet of things, simulation and virtual reality, augmented reality, big data, digital repositories, smart cities, ethical challenges, and socio-technical and cultural issues, including resistance to change. Additionally, at the end of the programs or electives, a publication could be established as a deliverable that could be later submitted to peer-reviewed journals [50]. Writing in English could be a challenge for Latin American researchers, but publishing helps one’s chances of getting future funding and helps build new multidisciplinary collaborations [50].

International and national funding agencies could play a key role in supporting interoperable, scalable projects at Latin American academic and research institutions. An important message to spread is that policymakers, funding agencies, and donors should stop funding isolated or stand-alone projects and initiatives. Skill development should be viewed as a continuous process discussed among stakeholders, including the Ministry of Health, academia, and private institutions [51,52].

e-Learning programs could help communication, provide knowledge acquisition for large numbers of trainees, and allow access to up-to-date knowledge to any person [10]. It could be an excellent resource for people with disabilities or those who are in remote or rural areas. Health care hackathons have also been reported as a reliable source of solutions to health care challenges, boosting the process of “co-creation” [53], and should be organized and evaluated in Latin American countries. Future studies of educational programs on digital health in the region could add valuable insights about training needs at different levels (decision-makers, digital health implementers, and users), the profile of trainees (including leadership skills), and highlight the urgent need for monitoring, evaluating, and publishing the lessons learned of those experiences.

Evidence from developing countries has shown the benefits of digital tools for strengthening health systems and improving decision-making by providing access to digital health training content, better support with health professional networks, and better case detection using disease surveillance systems [30], even in real-time [54]. Capacity-building programs should be viewed as a complex intervention and need proper funding, a network of colleagues, a clear career path, a multidisciplinary team, financial incentives, and a political commitment to support it and sustain [55,56]. Evaluation could be difficult, and they should consider context and how long after the program is initiated should the evaluation be started [55,56].

One of the biggest challenges is to ensure the sustainability and continuity of digital health initiatives, which should be documented with proper scientific evidence to raise awareness among decision-makers [30]. As Long et al pointed out [30]:

Large-scale or nationwide coverage of digital health interventions to support health workforce development is still rarely reported in the literature.

We would like to see more academic projects that become national digital health programs, such as Wawared in Peru [57-61].

Finally, Long et al proposed key areas of digital health research [30] for low- and middle-income countries, such as: (1) the value of scaling up digital health approaches to human resources for health management and support; (2) better evidence on the return of investment of digital health; (3) the effect of current donor and government procurement policies on scale-up of digital health technologies for health workforce development; and (4) the role of the private sector and philanthropists in digital health.

We hope to inspire and encourage Latin American decision-makers, innovators, and researchers to build together on the work already done through digital health solutions (eg, artificial intelligence, big data analytics) and find innovative ways for public and private partnerships to improve health workforce capacity. We also want them to focus on accelerating the pace at which they can further improve the health of populations.

## Abbreviations

AMIA: American Medical Informatics Association
CYTED-RITMOS: The Ibero-American Network of Mobile Technology and Health
eGCC: e-Government Cooperation Center
eHealth: electronic health
GPP: Global Partnership Program
HIMSS: Healthcare Information and Management Systems Society
HL7 LATAM: Health Level Seven Latin America
ICD: International Classification of Diseases
IMIA: International Medical Informatics Association
IMIA-LAC: Federation of Health Informatics for Latin America and the Caribbean
ITGH: Informatics Training for Global Health
mHealth: mobile health
PAHO: Pan American Health Organization
RECAINSA: The Central American Health Informatics Network
RELACSIS: The Latin American and Caribbean Network for Strengthening Health Information Systems
WHO: World Health Organization

## References

[1] Labrique A, Vasudevan L, Mehl G, Rosskam E, Hyder AA (2018). Digital Health and Health Systems of the Future. Glob Health Sci Pract.

[2] Vincens N, Emmelin M, Stafström Martin (2018). Social capital, income inequality and the social gradient in self-rated health in Latin America: A fixed effects analysis. Soc Sci Med.

[3] (2019). United Nations Development Programme.

[4] Farach N, Faba G, Julian S, Mejía Felipe, Cabieses B, D'Agostino M, Cortinois AA (2015). Stories From the Field: The Use of Information and Communication Technologies to Address the Health Needs of Underserved Populations in Latin America and the Caribbean. JMIR Public Health Surveill.

[5] Pan American Health Organization (2019). PAHO Iris.

[6] Malaquias RF, Albertin AL (2018). Challenges for development and technological advancement. Information Development.

[7] Novillo-Ortiz D, Dumit EM, D'Agostino M, Becerra-Posada F, Kelley ET, Torrent-Sellens J, Jiménez-Zarco Ana, Saigí-Rubió Francesc (2018). Digital health in the Americas: advances and challenges in connected health. BMJ Innov.

[8] (2014). World Health Organization.

[9] Luna D, Almerares A, Mayan JC, González Bernaldo de Quirós Fernán, Otero C (2014). Health Informatics in Developing Countries: Going beyond Pilot Practices to Sustainable Implementations: A Review of the Current Challenges. Healthc Inform Res.

[10] Otero P, Perrin C, Geissbulher A, Cheung N, Theera-Ampornpunt N, Lun K (2013). Informatics Education in Low-Resource Settings. Informatics Education in Healthcare.

[11] Novillo-Ortiz D, D'Agostino M, Becerra-Posada F (2016). Role of PAHO/WHO in eHealth Capacity Building in the Americas: Analysis of the 2011-2015 period. Rev Panam Salud Publica Aug.

[12] D´Agostino M, Mejía F, Martí M, Novillo-Ortiz D, Hazrum F, de CF (2017). Infoxication in health. Health information overload on the Internet and the risk of important information becoming invisible [in Spanish]. Rev Panam Salud Publica.

[13] Hersh W, Margolis A, Quirós Fernán, Otero P (2010). Building a health informatics workforce in developing countries. Health Aff (Millwood).

[14] Otero P, Hersh W, Luna D, González Bernaldo de Quirós F (2018). A Medical Informatics Distance-learning Course for Latin America. Methods Inf Med.

[15] Curioso WH, García PJ, Castillo GM, Blas MM, Perez-Brumer A, Zimic M, QUIPU R (2010). Strengthening global health informatics research within the andean region through international collaboration [in Spanish]. Rev Peru Med Exp Salud Publica.

[16] dos Santos Alaneir de Fátima, Alves HJ, Nogueira JT, Torres RM, Melo MDCB (2014). Telehealth distance education course in Latin America: analysis of an experience involving 15 countries. Telemed J E Health.

[17] Margolis A, Joglar F, de Quirós Fernán González Bernaldo, Baum A, Fernández Antonio, García Sofía, Arredondo AL, Hersh WR (2013). 10x10 comes full circle: Spanish version back to United States in Puerto Rico. Stud Health Technol Inform.

[18] Garcia NCG, Sarría EPG (2012). Medical Informatics in the Medical curriculum in Cuba [in Spanish]. RCIM.

[19] Mendoza Rojas HJ, Placencia Medina MD (2017). Use of information and communication technologies as teaching material in Human Medicine [in Spanish]. Investigación en Educación Médica.

[20] Piette JD, Valverde H, Marinec N, Jantz R, Kamis K, de la Vega CL, Woolley T, Pinto B (2014). Establishing an independent mobile health program for chronic disease self-management support in bolivia. Front Public Health.

[21] Blas MM, Curioso WH, Garcia PJ, Zimic M, Carcamo CP, Castagnetto JM, Lescano AG, Lopez DM (2011). Training the biomedical informatics workforce in Latin America: results of a needs assessment. BMJ Open.

[22] Villena J, Yoshiyama C, Sánchez Javier E, Hilario N, Merin L (2011). Prevalence of diabetic retinopathy in Peruvian patients with type 2 diabetes: results of a hospital-based retinal telescreening program. Rev Panam Salud Publica.

[23] Whitehead E, Dorfman V, Tremper G, Kramer A, Sigler A, Gosman A (2012). Telemedicine as a Means of Effective Speech Evaluation for Patients With Cleft Palate. Annals of Plastic Surgery.

[24] Chan MC, Bayer AM, Zunt JR, Blas MM, Garcia PJ (2018). Kuskaya: A training program for collaboration and innovation in global health. JMDH.

[25] (2013). AMIA.

[26] Detmer DE, Shortliffe E, Brown B (2010). AMIA.

[27] Quirós Fernán Gonzalez Bernaldo de, Baum A, Lira A (2019). Active Participation and Engagement of Residents in Clinical Informatics. Appl Clin Inform.

[28] Rodrigues RJ, Risk A (2003). eHealth in Latin America and the Caribbean: development and policy issues. J Med Internet Res.

[29] LeRouge CM, Gupta M, Corpart G, Arrieta A (2019). Health System Approaches Are Needed To Expand Telemedicine Use Across Nine Latin American Nations. Health Aff (Millwood).

[30] Long L, Pariyo G, Kallander K (2018). Digital Technologies for Health Workforce Development in Low- and Middle-Income Countries: A Scoping Review. Glob Health Sci Pract.

[31] Jayasuriya R (1999). Managing information systems for health services in a developing country: a case study using a contextualist framework. International Journal of Information Management.

[32] Odhiambo-Otieno GW (2005). Evaluation of existing district health management information systems a case study of the district health systems in Kenya. Int J Med Inform.

[33] Lima-Toivanen M, Pereira RM (2018). The contribution of eHealth in closing gaps in primary health care in selected countries of Latin America and the Caribbean. Rev Panam Salud Publica.

[34] Gerber T, Olazabal V, Brown K, Pablos-Mendez A (2010). An agenda for action on global e-health. Health Aff (Millwood).

[35] (2010). The Rockefeller Foundation.

[36] Curioso WH, Mechael PN (2010). Enhancing 'M-health' with south-to-south collaborations. Health Aff (Millwood).

[37] Curioso WH, Fuller S, Garcia PJ, Holmes KK, Kimball AM (2010). Ten years of international collaboration in biomedical informatics and beyond: the AMAUTA program in Peru. J Am Med Inform Assoc.

[38] Curioso WH, Hansen JR, Centurion-Lara A, Garcia PJ, Wolf FM, Fuller S, Holmes KK, Kimball AM (2008). Evaluation of a joint Bioinformatics and Medical Informatics international course in Peru. BMC Med Educ.

[39] The Andean Global Health Informatics Research and Training Center [in Spanish].

[40] Garcia PJ, Egoavil MS, Blas MM, Alvarado-Vásquez E, Curioso WH, Zimic M, Castagnetto JM, Lescano AG, Lopez DM, Carcamo CP (2015). Development and institutionalization of the first online certificate and Master Program of Biomedical Informatics in global health in Peru [in Spanish]. Rev Peru Med Exp Salud Publica.

[41] Espinosa A (2018). IMIA LAC. Yearb Med Inform.

[42] (2019). International Medical Informatics Association.

[43] (2019). RECAINSA.

[44] (2019). OPS.

[45] Saigí-Rubió F, Novillo-Ortiz D, Piette J (2017). CYTED-RITMOS network: toward the search for solutions to promote mobile health in Latin America [in Spanish]. Rev Panam Salud Publica May 25.

[46] HL7 LATAM.

[47] (2017). HIMMS Latin America.

[48] e-Government Cooperation Center (eGCC).

[49] (2019). World Health Organization.

[50] Garcia PJ, Curioso WH (2008). Strategies for aspiring biomedical researchers in resource-limited environments. PLoS Negl Trop Dis.

[51] Curioso WH, Espinoza-Portilla E (2015). Marco conceptual para el fortalecimiento de los sistemas de información en salud en el Perú. Rev Peru Med Exp Salud Publica.

[52] Curioso WH (2014). [eHealth in Peru: implementation of policies to strengthen health information systems]. Rev Panam Salud Publica.

[53] Olson KR, Walsh M, Garg P, Steel A, Mehta S, Data S, Petersen R, Guarino AJ, Bailey E, Bangsberg DR (2017). Health hackathons: theatre or substance? A survey assessment of outcomes from healthcare-focused hackathons in three countries. BMJ Innov.

[54] Curioso WH, Karras BT, Campos PE, Buendia C, Holmes KK, Kimball AM (2005). Design and implementation of Cell-PREVEN: a real-time surveillance system for adverse events using cell phones in Peru. AMIA Annu Symp Proc.

[55] Catwell L, Sheikh A (2009). Evaluating eHealth interventions: the need for continuous systemic evaluation. PLoS Med.

[56] Trostle J (1992). Research capacity building in international health: Definitions, evaluations and strategies for success. Social Science & Medicine.

[57] Curioso WH, Roman H, Perez-Lu J, Castagnetto JM, García Patricia J (2010). [Improving maternal health information systems: validation of electronic medical records in Callao, Peru]. Rev Peru Med Exp Salud Publica.

[58] Pérez-Lu JE, Iguiñiz Romero R, Bayer AM, García PJ (2015). Reduciendo las inequidades en salud y mejorando la salud materna mediante la mejora de los sistemas de información en salud: Wawared Perú. Rev Peru Med Exp Salud Publica.

[59] (2914). CEPAL.

[60] Pérez-Lu Jose E, Bayer A, Iguiñiz-Romero Ruth (2018). Information = equity? How increased access to information can enhance equity and improve health outcomes for pregnant women in Peru. J Public Health (Oxf).

[61] Curioso WH, Henríquez-Suarez Milagro, Espinoza-Portilla E (2018). [From Alma-Ata to the digital citizen: towards a digital primary health care in Peru]. Rev Peru Med Exp Salud Publica.

